# “But This Is the New Reality, and I Will Adapt”: Understanding Lecturers’ Experiences of COVID-19 Lockdown Online Learning and Teaching

**DOI:** 10.1007/s40670-023-01925-6

**Published:** 2023-10-31

**Authors:** Lynette J. van der Merwe, Sanet van Zyl, Gina Joubert

**Affiliations:** 1 Division Health Sciences Education, Office of the Dean, Faculty of Health Sciences, University of the Free State, PO Box 339, Bloemfontein, 9300 South Africa; 2 Department of Basic Medical Sciences, Faculty of Health Sciences, University of the Free State, PO Box 339, Bloemfontein, 9300 South Africa; 3 Department of Biostatistics, Faculty of Health Sciences, University of the Free State, PO Box 339, Bloemfontein, 9300 South Africa

**Keywords:** Blended learning, COVID-19, Educational approach, Online learning, South Africa, Technology

## Abstract

Following the 2020 COVID-19 pandemic national lockdown in South Africa, the University of the Free State launched various support initiatives for academic staff and students. Teaching and learning activities and assessments were adapted for emergency remote teaching. Students and academic staff members experienced disruption due to the migration to the online environment. This study aimed to investigate the experiences of academic staff members in an undergraduate medical programme using a mixed-methods approach in the form of a sequential exploratory design in two phases. Quantitative data were obtained through an online questionnaire survey that were triangulated and complemented with qualitative data obtained from responses to open questions in the questionnaire survey and online reflective essays. Quantitative data revealed that although most academic staff members had received training in and used mostly administrative functions in the learning management system (Blackboard) prior to lockdown, its uses almost doubled during the lockdown. Qualitative data analysis gave an in-depth understanding of academic staff members’ experiences identified in the themes Teaching and Learning, Assessment, Technology, Communication, and Personal Experience. Concerns were expressed regarding students’ access to technology and adaptation to online learning, and training needs and challenges were identified. The lessons learnt through the resilient, transformative responses to this global disruptor can guide future strategies for medical education.

## Introduction

On the 5th of March 2020, the first case of COVID-19 was confirmed in South Africa [1]. Subsequently, the South African Government announced a national lockdown starting on the 26th of March 2020. In response to this announcement and considering the unique realities facing higher education students, which include limited access to computers, devices and high-quality internet off-campus, the University of the Free State (UFS), located in central South Africa, launched various support initiatives for academic staff and students. Academic staff members who are responsible for student training (learning, teaching and assessment) had to quickly adapt teaching and learning activities and assessments for emergency remote teaching to ensue. Despite these initiatives, students and academic staff members experienced disruption in all aspects of their academic and personal life through this sudden transition.

Medical education relies mainly on face-to-face and some blended learning (e.g. clinical skills training and work-based learning). Migration to a purely online mode is not simple, as not all immersive learning activities are suited for the online environment. Online educational approaches using learning management systems, e.g. Blackboard, have been shown to be acceptable with varying perspectives among students and staff [2] and may be dependent on academic staff members’ experience and attitudes [3]. Online learning promotes student self-regulation, life-long learning, and other graduate attributes, but it may be more demanding. Coupled with social isolation as required during COVID-19, it may be a less comprehensive learning experience unless carefully planned [4]. Students do not adapt seamlessly to online learning [4]. The digital divide among students with varied socio-economic backgrounds in the South African higher education landscape further affects students’ learning experience and academic progress.

The sudden transition to online teaching, learning and assessment in higher education has influenced undergraduate students and academic staff members responsible for learning, teaching and assessment in the Faculty of Health Sciences in numerous ways. While a challenge is always a door to opportunity and innovation, the disruptive effects of the global pandemic may also be disastrous for some [5]. The COVID-19 pandemic has tested healthcare professionals, health sciences students and all those involved in training future healthcare professionals in every aspect of professional and personal life [6].

This study aimed to investigate academic staff members’ experiences of online learning and teaching in the undergraduate medical programme at the UFS during the COVID-19 pandemic lockdown in 2020. Understanding how lecturers dealt with a critical global disruptor should inform future planning and management.

## Method

We used a mixed-methods approach in the form of a sequential exploratory design in two phases [7]. The rationale for using mixed-methods flows from a pragmatist paradigm [8]. The advantage of mixed-methods research is that it draws on the strengths of quantitative and qualitative approaches [7]. The disruptive impact following the COVID-19 pandemic lockdown in 2020 can best be understood using diverse and comprehensive methods, especially in the complex medical education environment. Quantitative data from the questionnaire survey (phase 1) described the population and their experiences. These data were triangulated and complemented with qualitative data obtained from more than one source (responses to open questions in the questionnaire survey and reflective essays (phase 2) to contribute to the validity of findings and depth of understanding.

The undergraduate medical programme (MBChB) at the UFS is divided into three phases. Phase I (semester 1) and phase II (semesters 2–5) comprise preclinical and phase III (semesters 6–10) clinical training, respectively. The UFS uses the Blackboard online learning management system. The UFS Centre for Teaching and Learning (CTL) provides training and support for academic staff members responsible for student training (learning, teaching and assessment), using face-to-face and online training platforms. All the full-time employed academic staff members involved in the undergraduate MBChB programme in the Faculty of Health Sciences were included in the sample population. Faculty academic staff members include those appointed at the UFS and the joint employment establishment (UFS and Free State Department of Health). Faculty academic staff have medical or scientific qualifications (for example, general medical practitioners, medical specialists, and biomedical scientists) and are responsible for lecturing and clinical training.

The total population was estimated at 70. Academic staff members who were not involved in the transition to online teaching, learning and assessment in the MBChB programme during COVID-19 were excluded from the study population.

In phase 1 of the study (conducted from July to August 2020), academic staff members in the Faculty of Health Sciences involved in student training in the MBChB programme (undergraduate medical programme) were invited to complete anonymous online questionnaires distributed electronically via e-mail using the Evasys online automated survey platform (https://evasys.de/en/). The questionnaire included closed and open questions obtaining quantitative and qualitative data, investigating academic staff members’ experiences of online teaching, learning and assessment during the COVID-19 pandemic lockdown period. The e-mail invitation included information about the study. Consent was inferred through the completion of the questionnaire. The questionnaire was available for 2 weeks initially; after that, a further reminder was sent, and the questionnaire was available for an additional 2 weeks.

Phase 2 took place 3 months after phase 1 (November 2020). We investigated academic staff members’ personal experiences of online teaching, learning and assessment in the MBChB programme during the 2020 COVID-19 pandemic lockdown through an online anonymous reflective essay (maximum 500 words). A link to an Evasys survey platform was provided to academic staff in an e-mail invitation to ensure anonymous submission. No personal identifiers were requested from participants besides indicating in what phase of the programme they are involved.

The e-mail invitation was sent to all academic staff members once a week for 4 weeks, with a submission deadline set for one 1 after the final reminder date. All reflective essays submitted during 5 weeks were included for analysis. Consent was assumed through voluntary participation.

All data were handled with strict confidentiality. Statistical analysis of quantitative data was done by one of the researchers, using SAS Version 9.4. Descriptive statistics, namely frequencies and percentages of categorical data, were reported. The statistical significance of differences in responses regarding training and activities before and during the COVID-19 lockdown period was assessed using McNemar’s test for paired data. Internal validity was addressed by ensuring a well-structured questionnaire and doing a pilot study. Qualitative data were analysed using thematic analysis. Through an inductive process, themes were established from the data. The themes were determined by reading the qualitative data (responses to open questions and reflective essays) and developing a coding scheme. One researcher identified themes in an initial round of analysis, while a second researcher subsequently read the qualitative data and themes to further refine the analysis. Two researchers in the team agreed on the final codes and themes. Thematic analysis allows for identifying patterns (themes) in the data and provides breadth and depth to understanding and interpreting underlying issues [9]. Thematic analysis of narrative reflections provides rich descriptions of personal experiences [10, 11].

The Health Sciences Research Ethics Committee of the University of the Free State approved the study (UFS-HSD2020/1112/2909) and relevant UFS Gatekeepers’ approval was obtained.

## Results

We report general results, including the characteristics of participants (academic staff members), descriptive statistics of quantitative data (phase 1) and thematic analysis of qualitative data (phases 1 and 2).

### Characteristics of Participants

Of the target population of 70 academic staff members, 36 full-time employed academic staff members participated in phase 1 of the study (response rate 51.4%), while 12 participated in phase 2 by writing online reflections (response rate 17.1%). One participant who responded in phase 2 but did not write a reflection was excluded from the analysis. In phase 1, 20 participants (55.6%) were employed by the UFS, and 16 (44.4%) were employed on the joint employment of two establishments (UFS and Free State Department of Health). Fifteen (41.7%) participants indicated that they had occupied the current position for 11 years or longer, while 16 (44.4%) indicated that they had been in the position for 5 years or less. In phase 2, similar background information was not requested.

In phase 1, nine (25.0%) academic staff members indicated involvement in phase I, 23 (63.9%) in phase II, and 22 (61.1%) in phase III of the MBChB programme (ranging between one and eight modules). Most participants (*n* = 31, 88.6%) fulfilled administrative roles in two or more modules. In phase 2, six academic staff members were involved in teaching and learning in phase II of the MBChB programme, three in phase III, and one in phase I. Five academic staff members were involved in teaching and learning in other Schools in the Faculty of Health Sciences (excluding the MBChB programme).

### Quantitative Data: Phase 1

Questions determined academic staff members’ use of various online training activities before and during the COVID-19 lockdown period (illustrated in Table 1). Twenty-four participants (66.7%) indicated that they had received training on the use of Blackboard administrative functions before the lockdown period, and 11 (30.6%) received training during the 8 months since the commencement of the lockdown period in March 2020 (*p* < 0.001). Statistically significantly more participants reported receiving training in online teaching (*n* = 23, 63.9%; *p* = 0.001), formative (*n* = 21, 58.3%; *p* = 0.018) and summative assessment (*n* = 20, 55.6%; *p* = 0.001) during the lockdown compared to before the lockdown. Significantly more participants indicated that they had already received training in online assessment modalities before lockdown (QuestionMark: *n* = 18, 50.0%; *p* = 0.003).

**Table 1:** Participants’ Blackboard and online assessment training before and during the COVID-19 lockdown

**Training activity**	**Before (*N* = 36)**	**During (*N* = 36)**	***p*-value**
	***n* (%)**	***n* (%)**	
Blackboard administration	24 (66.7)	11 (30.6)	0.001
Online teaching	11 (30.6)	23 (63.9)	0.001
Formative assessment	11 (30.6)	21 (58.3)	0.018
Summative assessment	5 (13.9)	20 (55.6)	0.001
QuestionMark	18 (50.0)	6 (16.7)	0.003
Respondus	4 (11.1)	3 (8.3)	0.655
Other (Self-training, Blackboard Collaborate)	3 (8.3)	4 (11.1)	0.564

Table 2 indicates that academic staff members increased the use of all Blackboard activities during the 2020 COVID-19 lockdown period. Blackboard class announcements increased from 24 (66.7%) before to 28 (77.8%) during the lockdown (*p* = 0.157), while Blackboard Collaborate use for presenting lectures increased from five (13.9%) to 33 (91.2%), *p* < 0.001. Academic staff members using summative assessment (6 (16.7%) to 11 (30.6%); *p* = 0.096) doubled and the increase in use of formative assessments from (10 (27.8%) to 21 (58.3%); *p* = 0.002), and grading on Blackboard (10 (27.8%) to 20 (55.6%); *p* = 0.002) were statistically significant. A few academic staff members indicated that they started using other activities (journals, discussion boards, blogs) during the lockdown, but these percentages were not statistically significantly higher than before the lockdown. No academic staff members reported using Wikis before or during the lockdown.

**Table 2:** Blackboard activities utilised by participants before and during the COVID-19 lockdown

**Blackboard activity**	**Before (*N* = 36)**	**During (*N* = 36)**	***p*-value**
	***n* (%)**	***n* (%)**	
Announcements	24 (66.7)	28 (77.8)	0.157
Blackboard Collaborate	5 (13.9)	33 (91.2)	< 0.001
Formative assessment	10 (27.8)	21 (58.3)	0.002
Summative assessment	6 (16.7)	11 (30.6)	0.096
Grading	10 (27.8)	20 (55.6)	0.002
Journals	1 (2.8)	4 (11.1)	0.083
Discussion boards	0 (0.0)	3 (8.3)	0.083
Blogs	0 (0.0)	1 (2.8)	0.317
Wikis	0 (0.0)	0 (0.0)	

Table 3 indicates academic staff members’ experience of implementing online teaching and learning activities during the lockdown. Most academic staff members indicated that they found it easy or average to do activities including build their module page online, source material for online teaching, engage with students on the learning platform, bring prior learning into the learning environment, upload material to meet the learning needs of students, scaffold learning materials and activities for students, use Blackboard Collaborate, set up assignments, set up formative assessments, use the Grade function, and have 80% response to assignments. Most academic staff members found it average or difficult to have 80% online participation of students or 80% response to tests/assignments. Most academic staff members reported that using learning activities, e.g. journals, discussion boards and blogs, self-reflection, and peer evaluation was not applicable to them.

**Table 3:** Implementation of online teaching and learning activities during the COVID-19 lockdown

**Response to “How easy was it for you to…”**	**Difficult**	**Average**	**Easy**	**N/A**
	***n* (%)**	***n* (%)**	***n* (%)**	***n* (%)**
Build the module page online (*n* = 30)	4 (13.3)	12 (40.0)	14 (46.7)	-
Source learning material for online teaching (*n* = 31)	4 (12.9)	16 (51.6)	11 (35.5)	-
Use learning activities: journals, discussion boards, blogs (*n* = 31)	6 (19.4)	3 (9.7)	4 (12.9)	18 (58.1)
Use self-reflection and/or peer-evaluation (*n* = 31)	6 (19.4)	6 (19.4)	2 (6.5)	17 (54.8)
Engage with students on the learning platform (*n* = 31)	6 (19.4)	13 (42.0)	12 (38.7)	-
Bring prior learning into the learning environment (*n* = 30)	3 (10.0)	17 (56.7)	10 (33.3)	-
Source audio-visual material (video’s, TEDtalks) (*n* = 30)	8 (26.7)	14 (46.7)	8 (26.7)	-
Upload material to meet the learning needs of students (*n* = 30)	1 (3.3)	11 (36.7)	18 (60.0)	-
Scaffold learning materials and activities for students (*n* = 30)	3 (10.0)	19 (63.3)	8 (26.7)	-
Host synchronous online sessions (*n* = 30)	3 (10.0)	9 (30.0)	9 (30.0)	9 (30.0)
Have 80% online participation of students (*n* = 29)	10 (34.5)	12 (41.4)	7 (24.1)	-
Use Blackboard Collaborate (*n* = 30)	8 (26.7)	10 (33.3)	10 (33.3)	-
Set up assignments (*n* = 30)	3 (10.0)	12 (40.0)	7 (23.3)	8 (26.7)
Set up formative assessment (*n* = 29)	2 (6.9)	11 (37.9)	12 (41.4)	4 (13.8)
Use Grade function (*n* = 30)	1 (3.3)	11 (36.7)	11 (36.7)	7 (23.3)
Have an 80% student response to tests/assessments (*n* = 30)	10 (33.3)	13 (43.3)	7 (23.3)	-
Have an 80% response to assignments (*n* = 30)	1 (3.3)	10 (33.3)	8 (26.7)	11 (6.67)
Include COVID-19 into learning material (*n* = 30)	4 (13.3)	4 (13.3)	10 (33.3)	12 (40.0)

N/A, not applicable

### Thematic Analysis: Phases 1 and 2

Analysis of qualitative data collected from phase 1 (responses to open questions) and phase 2 (online reflective essays) was done concurrently, providing corroboration of findings. The data are available in Appendices A and B.

Qualitative data collected during phase 1 (36 participants) included responses to open questions. These included “What were the major challenges for you to transition to online teaching?” (34 responses, two missing), “What were your most positive experiences of the transition to online teaching?” (32 responses, four missing), and “What are your suggested training needs?” (28 responses, eight missing). Qualitative data collected during phase 2 (11 participants) were in the form of a reflective essay on their experience of transitioning to online learning during the COVID-19 lockdown in 2020.

Five themes and related categories emerged from qualitative analysis. These included ***Teaching and Learning*** (Engagement, Online approach, Responsibility), ***Assessment*** (Integrity, Convenience), ***Technology*** (Access, Support), ***Communication*** (Platforms, Frequency), and ***Personal experience*** (Remote setting, Growth, Adaptability). Illustrative quotes are provided from the responses to open questions in the survey (S + participant number) and reflective essay (R + participant number).

#### Teaching and Learning

Engagement emerged as a prominent category in the theme ***Teaching and Learning***. Academic staff members acknowledged that adapting from highly interactive face-to-face settings to an online format was challenging and that the training provided by the CTL was useful to equip them:

*Engagement with the students – at times I feel I am talking to myself (S3)*

*Despite all this effort – student engagement with regards to BBC*^1^*discussions was minimal at best even with polling and MCQs*^2^*being availed throughout the sessions….I could not tell if the majority were even ‘in’ the session – or had just simply logged in for the attendance register and left the room to attend to other activities.* (S28)

Academic staff members expressed the need for training in improving student engagement online, e.g. “I need further assistance in conforming my lectures to be more interactive” (S9).

Another category, the online approach, revealed that while this approach offered opportunities for enhanced teaching and learning, it was also limited by academic staff members’ perceptions:

*Having to move over to a completely online environment …. has been quite a challenge. This has however also provided the opportunity for growth and learning on how to adapt to using the online platform to support students and facilitate learning in a way that is acceptable and understandable to students. *(R5)

This is illustrated by quotes such as “very impersonal and not conducive to training” (S24), “my modules are better learned physically than online” (S17), and “I am yet to be convinced that practical clinical skills can be better taught online rather than in-person” (S8). Even the positive aspects of online learning and teaching (“unrestricted access to the learning material” (S28), “you only have to give a lecture once” (S30), and “students tend to receive more individual attention when using the online teaching platform” (S21)) and the fact that “online learning benefits the students who are very self-motivated to learn” (S13) may be negated in the light of it being “an obstacle for those students who benefit from the sense of community that face-to-face learning offers” (S13).

The responsibility of lecturers and students was highlighted as another category in this theme. Lecturers felt that online sessions encouraged only students who were genuinely interested in engaging. While some felt that it reduced students’ responsibility for their learning (“I think most of the time the lectures over-communicated to the student and it made them lazy to read instructions and the phase guide. They wanted to be spoon feed even more” (R2)) others indicated that students “took more responsibility for their own learning” (S7) although students’ familiarity with the online approach limited their progress (“I do worry that because students aren’t used to online studying they stay behind even with all our efforts” (R11)).

#### Assessment

Integrity emerged as a category in the theme ***Assessment***. Lecturers mentioned that students used “digitally assisted platforms to complete online tests and that many students would subsequently lack foundational theoretical knowledge to thrive under high-pressure face-to-face situations” (R1) and cheated by using notes and other resources so that maintaining test security was a challenge. While there was less work marking with online assessments, it took more time to prepare and set up online assessments. Although some lecturers indicated especially preclinical students having better marks with online assessments, others stated a decline in student achievement (“Marks have fallen dramatically” (S29)), and concern about foundational knowledge acquisition as shown above. There was a need for increased training in setting up online tests and assessments (“I would like more personal interaction with blackboard personal in the way to set up online tests and assessments- the broad outlines were not helpful for me in the way that I need to evaluate the students” (S12)) and methods to “appraise and audit online tools” (S15).

#### Technology

Under this theme, access for both students and academic staff to use the variety of online options emerged as a category. Connectivity and technical issues and variable degrees of familiarity when using electronic media or the learning management system impacted on the use of technology (“In the beginning was uncertain, not sure if it was effective worried about all the IT and techno problems that might occur during an online class” (R2)). In some cases, lecturers supported students by providing access or resources (“I felt like I was doing my work and expected to do that of the students as well- as students were also indicating that they did not have access to various resources off campus- so I had to bridge that gap for them” (S28)) and reported concern that students would be “left behind” (R12) without the necessary access and support.

Another category in the theme ***Technology*** was support. Lecturers expressed concerns about the initial rollout of support for students in the early stage of the lockdown:

*Although the university tried to develop systems of communication and connectivity a lot of students especially from disadvantaged backgrounds could not get access and were ‘left behind’. The Global Protect App did not work students did not get the data that was promised. The roll out of computers did not go well. NSFAS*^3^*promised to provide students with laptops but up until now that has not happened. Not being able to get access the campus was very difficult because lecturers and students had left their resources behind.* (R12)

Lecturers also expressed appreciation for the support provided by the institutional CTL and the Faculty of Health Sciences to academic staff and students during the transition. This is illustrated in statements such as “amount of training made available to make this transition easier” (S32), “The immense support offered by CTL to assist staff in creating the online platform” (S23) and “In this strange times I believe that the students were assisted in the best way possible. Congratulations to the Health Faculty” (R9). Lecturers expressed the need for continuous and further training, especially in using the online learning platform and optimising interaction with students. This is supported by statements such as “Continuous training in Blackboard features—I am only using a very small part of what it actually provides. I am probably not even aware of all the possibilities.” (S5).

#### Communication

Many academic staff members alluded to the sense of impaired communication due to the unfamiliarity of the online platform as opposed to seeing students in person. Statements like “Familiarizing myself with the concept of not seeing the student faces while giving a lecture. Not being sure if my point is going across as intended” (S5) and “Not able to evaluate the body language of the students – or to directly interact with students” (S16) as well as a sense of being unprepared for the sudden transition raised concerns about educational efficacy, lecturer-student relationships, and student wellbeing. Challenges were mentioned, including “To gauge where the students were academically and emotionally. To maintain the teacher-student bond” (S33).

As part of this theme, the frequency of communication and using alternative methods, e.g. e-mail and text messaging, emerged. One lecturer cautioned against the effect of over-communication in decreasing students taking responsibility for their learning:

*…Although there were no issues it was not enjoyable. Prefer to see the students face-to-face. I think most of the time the lectures over-communicated to the student, and it made them lazy to read instructions and the phase guide. They wanted to be spoon feed, even more, my impression. *(R2)

Although lecturers welcomed the engagement and positive feedback from students on e-mail communication, they also noted being inundated with “The ‘million’ e-mails and questions. I think it is much easier for students to ask questions behind an ‘unknown identity’” (S22) adding to their already full work schedules.

Using alternative communication platforms, e.g. WhatsApp, afforded flexibility and opened channels to stay in touch with students, and some lecturers preferred these platforms to the learning management system (“I found Blackboard difficult and cumbersome to navigate- not always sure whether what I planned would happen. sting announcements is much more complicated than just sending a whatsap [sic] message on a group for example” (S10)).

#### Personal Experience

Many academic staff members indicated how the changes due to the lockdown impacted their personal lives and experience of an uncertain new environment (“the feeling of not being in control” (S24)). This is illustrated in quotes such as “However- one had to stay self-motivated and constantly remind yourself that you are an essential piece of a puzzle and your contribution was needed” (S28) and “Challenging times but allowing for a lot of personal growth and reflection” (R7).

The remote setting emerged as a category under the theme ***Personal experience***. Although some academic staff members experienced the transition to online teaching as positive due to the convenience and flexibility of working remotely through creative, innovative methods (“we can successfully work from home in any case” (R11) and “being creative and innovative” (S3), and “I could teach from the comfort of my home. I could be contacted online anywhere at any time” (S12)), others experienced isolation and frustration. This is illustrated in quotes such as “It was very challenging to set up a work space connectivity and routine” (R11), “More often than not- I was working from 8:00 to 23:00- because one could simply not switch off working from home- constantly surrounded by work and an endless stream of e-mails from students- and work-related deadlines” (S28), and “my experience was the exact opposite and was made more difficult by feelings of isolation as someone who lives on their own” (S28).

Furthermore, the category personal growth and adaptability was identified through academic staff members mentioning how they dealt with the changing, unpredictable environment (“But this is the new reality and I will adapt” (R3)), seeing this as an opportunity for growth, reflection, development and innovation (“Learning new online teaching skills – and feeling more confident and empowered in that regard” (S15) and “new experiences and broadening of my horizon in online teaching” (S16)). The downside was mentioned in terms of time management, drained personal resources (“online teaching required me to fish out more money from my personal pocket to buy data to work online” (S9)) and increased responsibilities that did not yield recognition:

*It took a lot of additional time to listen to podcasts attend webinars and seek examples to ensure that the teaching and learning, as well as assessments, were good quality. Unfortunately, the additional time spent on all these activities did not necessarily reflect on our performance management and research outputs. *(R7)

Overall, despite acknowledging challenges, personal growth was evidenced through statements such as “resilience and being optimistic are character traits I have learnt master” (S28). One lecturer summarised their experience in this way:

*In general the university and the faculty did its best under the difficult circumstance to support staff and students. There is no doubt everybody did their best to deliver quality teaching and support students. There was a lot of innovation and creativity. Although the period was emotionally draining there was resilience among staff and students. *(R12)

## Discussion

COVID-19 lockdown in 2020 had an unprecedented impact on the world and the continuum of medical education, requiring rapid adjustments and most likely enduring educational developments [12]. At the UFS, the continuation of the undergraduate medical programme included adopting online educational delivery and transitioning to a blended learning approach with synchronous and asynchronous online theoretical training, in-person clinical placements and practical skills training adapted to COVID-19 regulations, and a combination of face-to-face and online assessments. This is congruent with descriptions of adaptations to clinical learning reported in a recent systematic review [13] and a scoping review on the impact of COVID-19 on medical education [5].

Academic staff members’ experience at the UFS highlighted the catalytic impact of the crisis to fast-track change and drive innovation [14]. While most academic staff members indicated familiarity with the Blackboard learning management system administrative functions through training and use, the transition to online learning sped up training interest, implementation of online teaching and assessment activities, and innovative, creative and practical problem-solving to ensure continuity in training and student support. Academic staff members utilised different training opportunities to familiarise themselves with the online environment and improve their skills. Although quantitative data indicated that academic staff members increasingly implemented online teaching and assessment activities and were comfortable doing so, triangulation of these findings with qualitative data exposed a more nuanced perspective, including concerns about the stability, feasibility, and accountability of enforced change. This is supported by literature calling for equity in socially just learning environments and fostering a growth mindset among healthcare professionals [14].

In a post-pandemic educational environment, we should reflect on the lessons learnt to optimise what is best about online approaches rather than merely sustaining the status quo [15]. Findings from this study revealed that regarding teaching and learning, student engagement, the online learning approach and shared responsibility among students and academic staff were essential considerations, as well as the integrity and convenience of assessments. Sound pedagogical approaches need meticulous planning, implementation review and oversight, all of which require time and expertise that were unavailable during the COVID-19 pandemic response [15]. However, paying attention to the evidence and issues highlighted by those at the “coalface” of implementing forced crisis-driven change should inform educational approaches.

Leveraging technology in learning cannot be done without addressing the inequality in access to and availability of essential resources, including infrastructure, devices, connectivity, data, and expertise. The findings from this study gave a thick description of the complex reality academic staff and students face in a resource-constrained environment and concur with the needs in lower- and middle-income countries highlighted by Connolly and Abdallah [5]. Furthermore, this study also emphasised that innovative and creative solutions to communication in the educational environment and fostering collaborative relationships between academic staff and students are necessary to ensure optimal delivery of medical education to prepare the next generation of healthcare workers for an increasingly volatile, uncertain, complex and ambiguous world [16].

Finally, the study yielded a layered understanding of academic staff members’ personal experience of growth and adaptability during a time of unprecedented uncertainty and emergent change. The COVID-19 pandemic will be recorded as a historical inflection point that not only tested global resources and resolve [14] but has underlined numerous skills necessary to be flexible and adaptable to change [16].

## Limitations

Although 51.4% of the academic staff members participated in the questionnaire survey, the low number of participants (*n* = 36) limits meaningful comparisons between and across groups as well as the generalisability of the findings. The low response rate to the online reflective essay in the second phase (17.1%) and the fact that these reflections were very short (median word count 113 words, range 14–396 words) were mitigated by concurrent thematic analysis of the rich qualitative data emerging from responses to open questions from 33 participants in phase 1.

## Conclusions

Overall, academic staff members responsible for learning, teaching and assessment of undergraduate medical students had positive experiences of online learning during the COVID-19 pandemic lockdown period, and it was perceived as challenging yet valuable. Concerns about students’ access to technology and adaptation to online learning were expressed. In-depth understanding of academic staff members’ experiences was identified in the themes *Teaching and Learning*, *Assessment*, *Technology*, *Communication* and *Personal Experience*.

The training needs and challenges identified from these findings should guide future strategic imperatives for medical education. By recognising and reinforcing resilient, transformative responses to this global disruptor, future generations can build on our lessons learnt.

## Data Availability

All data generated or analysed during this study are included in this published article (and its supplementary information files).

## Appendix A

### Qualitative Data Reflective Essay

(Quotes used in text highlighted in **bold**).

1. The lack of engagement from the students make the online platform challenging. My classes encourage interaction and debate between students and myself. I found this aspect of teaching missing with the online format. I found the students initially struggled with online assessments but as the semester progressed the marks became progressively skewed to the higher end of the spectrum. My personal opinion is that the students quickly learned to use digitally assisted platforms to complete online tests and that many students would subsequently lack foundational theoretical knowledge to thrive under high-pressure face-to-face situations. My main challenge is to find a balance in the online assessments between application of theories and the recollection of hard facts which is vital for many of the health sciences.
2. In the beginning was uncertain not sure if it was effective worried about all the IT and techno problems that might occur during an online class. But I received great support from Christopher from BlackBoard. It was great to talk to someone and not try to figure out the notes. The support made big difference. After two assessments that went quite smoothly I actually consider doing certain assessments permanently via online. Teaching online experience is very different. I prefer the face to face class. Although there were no issues it was not enjoyable. Prefer to see the students face to face. I think most of the time the lectures over-communicated to the student and it made them lazy to read instructions and the phase guide. They wanted to be spoon feed even more my impression.
3. I am involved in Phase II. This is a second semester module. We had time to record all the lectures ahead of time. I found it difficult to give an online lecture and manage the comments simultaneously. The students preferred to log into the lecture from home rather than being split into two lecture halls. It is difficult for me to give a 40 min lecture with very little interaction with the students. **But this is the new reality and I will adapt**. Will have to build in quizzes into the lectures to ensure interaction. The students seemed to do worse in the second test (which involves more integration than the first test).
  2) I gave two BSc(Hons) lectures at Stage 2 of the lockdown. There were just two students sometimes via social media and sometimes by email. One should rather take a few minutes longer and discuss decisions that affect a large numbers of people. The Rectorate very soon settled into the mode of managing the pandemic. One can see that the Vice-Chancellor is an engineer that knows how to manage large scale operations. In 2021 we will probably keep on recording lectures. Giving lectures online also cuts down commuting especially in the clinical years. I found Blackboard Collaborate frustrating. Microsoft Teams is a much easier medium. Zoom became better. ";
  "Two of my children are studying medicine. The one in Phase III found it frustrating not to get as much clinical exposure as he wanted to. How as the lockdown became lighter he found his feet. He caught all the babies he had to. He found Obstetrics and Gynaecology badly organised and dictatorial. The teaching co-ordinator there treats students in the heavy-handed way that students were treated in the 1970s. Their written exam was very badly set up form both language and educational points of view. The student in Phase II had difficultly in Level 5 lockdown but after that a group of them met in one of their parents' house to study together around a big dining room table. They kept a tight 08:00–16:00 schedule on weekdays and even on weekends when there were tests. I pity students that only had cellphones and very little data to 4keep in contact. However the UFS went out of its way to help them. We will see about the pass rates a the end of 25,020.
4. I only missed one lecture that I present every 14 days to 5th year students. I provided an illustrated text and feedback was positive. For a postgraduate course the examination and other portfolio tasks had to be moved to Teams and Black8board. It worked well and was mostly well received by students thou they did ask for an element of direct contact in the future.
5. **"Teaching during the COVD-19 pandemic has been challenging. Having to move over to a completely online environment with first-year students who do not necessarily have the experience and/or skill required for working completely online as well as the necessary infrastructure (for some) has been quite a challenge. This has however also provided the opportunity for growth and learning on how to adapt to using the online platform to support students and facilitate learning in a way that is acceptable and understandable to students.**
6. "Students in the School of Clinical Medicine appeared to adjust to the online learning environment much quicker and more effectively than students in the School of Health and Rehabilitation Sciences for reasons that are unclear to me.
7. ;"Challenging times but allowing for a lot of personal growth and reflection how to best engage with teaching and learning during this time. It was the first time that we utilised blended learning and I had to engage with other institutions departments to draw from their experience and examples to ensure that the programme met the set outcomes. It took a lot of additional time to listen to podcasts attend webinars and seek examples to ensure that the teaching and learning as well as assessments were good quality. Unfortunately the additional time spent on all these activities did not necessarily reflect on our performance management and research outputs. Personal time in the evenings merged into working hours just to make sure that all is covered and time was spent on voice overs but it was also most unfortunate that all staff members did not equally participate to fine solutions and assist with new methods. The good is that we have now learned that the students actually did a lot better if they were allowed to study in their own time frames rather than attend face 2 face classes all the time and that they took more responsibility for their own learning. A lessons to be learned. We are going to continue with blended learning in the future.";"";
8. (*no reflection*).
9. 9. The voice over on power point was for me to one dimensional. Face-to-face through video or zoom would have been better. At the time I was pressed for time.";**"In this strange times I believe that the students were assisted in the best way possible. Congratulations to the Health Faculty".**
10. ;"I think it is an excellent method that should also be used in future.
11. I think if we were a little more prepared it would have been easier but that's life! Lockdown taught us a lot especially technologically. Being in a very practical field I think we have learnt that theory can safely be **taught I do worry that because students aren't used to online studying they stay behind even with all our efforts** (more extensive PPP's online tests for their own revision and not for marks after every theory section). This has a huge impact on our practicals if they have not done the theory yet on their own or if they don't practice the practical work at home as well as required.. But all in all I hope the powers that be have seen that we can successfully work from home in any case if we want to do online webinars 10 of us can't sit in open offices and do that impossible! The tests online made our marking much less but the students could definitely use their notes and did well (mostly). Personally I don't mind how they get to the theory answer with the application and insight questions their books won't help them and in a practical even if you know the question it is still all about your execution (our practical learning and assessment has still all been f2f). In lockdown I asked students to whatsapp me short video's of techniques that they perform which worked very well.
  I then gave feedback via whatsapp voice note or text. I will definitely do this again. But this year has been difficult and tiring and we can see that our junior years (1st and 2nd years) have not had the practical experience and practice that previous years have had.
12. ;"It was very challenging to set up a work space connectivity and routine. Had to rely on the university and faculty direction/guidance. There was concern about students who did not have the resources and were expected to perform. Although the university tried to develop systems of communication and connectivity a lot of students especially form disadvantaged backgrounds could not get access and were 'left behind'. The Global Protect App did not work students did not get the data that was promised. The roll out of computers did not go well. NSFAS promised to provide students with laptops but up until now that has not happened. Not being able to get access the campus was very difficult because lecturers and students had left their resources behind.";**"In general the university and the faculty did its best under the difficult circumstance to support staff and students. There is no doubt everybody did their best to deliver quality teaching and support students. There was a lot of innovation and creativity. Although the period was emotionally draining there was resilience among staff and students".**

## Appendix B

(Quotes used in text highlighted in **bold**).

### What were the Major Challenges for you to Transition to Online Teaching? (N = 34 + 2 Missing)

1. To change the format of lectures which were very interactive to a format that can be used for pure online teaching
2. A major challenge was finding ways to assess to students. The sessions held by CTL regarding assessments on Blackboard was very useful.
3. **Engagement with the students- at times i feel I am talking to myself.**
4. Ensuring that students were in fact engaging in the materials as they would with face to face sessions
  Designing the layout of the program and getting the materials ready
  Occasional internet connectivity or technical difficulties
  Ensuring fairness and equitable access for all students
5. **Familiarizing myself with the concept of not seeing the student faces while giving a lecture. Not being sure if my point is going across as intented**
6. Getting all the consultants to use Blackboard
  Uploading all the material is very time consuming
  Having to always be available as consultants need you on standby for all the hiccups that occur during online presentations
7. Getting connected at home Having students to access Global protect Getting access to campus and getting teaching material e.g. workbooks Having to rely on 1 person to upload material
8. Getting used to and comfortable with the new electronic media and the blended learning environment
9. Having to formulate new teaching strategies in terms of not having a specimen/cadaver physically present to teach the students.
  Not having F2F interaction with the students for discussion and teaching of the module content.
  **Online teaching required me to fish out more money from my personal pocket to buy data to work online**
10. **I found blackboard difficult and cumbersome to navigate- not always sure whether what I planned would happen.**
  **Posting announcements is much more complicated than just sending a whatsap message on a group for example.**
  I was not sure how many students would pithc up in real time for events scheduled on blackboard collaborate- interaction for me with the students on blackboard collab was more difficult as you have to wait for students to volunteer and can't call on students as I sometimes do in class.
  Connectivity for students in the beginning of lockdown was a challenge as well
11. I uploaded learning material as session presenter and dd not experience any challenges.
  I liaised with module leaders
12. Lack of a consistent framework
13. Never used it before
14. No challenges really- however i prefer face to face interaction with the students
15. No previous training or experience
16. **Not able to evaluate the body language of the students- or to directly interact with students**
17. Not having a technological background
18. Not having the personal (face-to-face) interaction with the students
  Non-respondent students
19. Remains temporary assistance not envisaged as permeant communication tool
20. Some students not having smart phones- data.
  Learning online platforms
21. Student interaction
  Answering questions
  Confirming whether they understand concepts before moving on to the next slide
22. Student participation was difficult in the beginning due to lack of resources for students at home.
  There were many helpful resources provided- but much of these came after the fact- and would have been useful earlier in the process
23. Support staff not available to find material.
  Working from hospital with poor internet connection
24. **The feeling of not being in control: especially in the beginning when planning changed frequently.**
   I was unsure how to present online and feared that I will make mistakes that will frustrate the whole class.
  Students were also more uncertain and there were many queries and questions to answer- especially in the beginning.
  From a practical point of view: scheduling of time-off: My vacation had to be postponed several times due to changes in work needs. I eventually scheduled my leave during a period when there were less lectures scheduled for me and continued with those lectures online. Online did however make it possible to take leave.
  I had to set up a home office buying a printer and sorting out data issues.
25. The interaction from the students decreased
26. The students had unequal access to high speed internet connections.
  They were somewhat overwhelmed by the amount of learning material and assignmenrts expected.
  The sheer amount of information necesary to read about adjustments in teaching and covid related research
27. The time it took to prepare traditional learning material to use in the online format. The time it took to prepare and set up e-assessments
28. The volume of work- e.g. the need for additional supportive materials- creating exercises to keep students engaged and excited about the module content- was overwhelming at times. **More often than not- I was working from 8:00 to 23:00- because one could simply not switch off working from home- constantly surrounded by work and an endless stream of emails from students- and work- related deadlines**. I would often feel guilty if I could not respond to all student inquiries on the day and found myself in the early hours of the morning- often sourcing additional material for students from the internet in the hopes of further clarifying difficult concepts.
  This is something which we would normally have required students to do for themselves in line with cultivating a spirit of independence and being more responsible and accountable for their own learning.
  I felt like I was doing my work and expected to do that of the students as well- as students were also indicating that they **did not have access to various resources off campus- so I had to bridge that gap for them.**
  **Despite all this effort- student engagement with regards to having BBC**^4^** discussions was minimal at best even with polling and MCQ's being availed throughout the sessions. It was always the same students who engaged- while I could not tell if the majority were even 'in' the session- or had just simply logged in for the attendance register and left the room to attend to other activities.**
  This experience was very draining energetically and mentally- but one had to somehow keep up the enthusiasm for the students sake- as we were often been reminded of what a difficult time this was for them- while negating the fact that so much more was now being required of the lecturers as well.
   It just felt like one had no excuse to feel overwhelmed because you were now working from the comfort of your home- **but my experience was the exact opposite- and was made more difficult by feelings of isolation as someone who lives on their own. However- one had to stay self- motivated and constantly remind yourself that you are an essential piece of a puzzle and your contribution was needed.**
  **Hence- resilience- and being optimistic are character traits I have learnt master**
29. Time it took to make audio visual presentations.
  Poor student insight.
  No bedside clinical teaching.
  The weak students slip through.
  **Marks have fallen dramatically**
30. Time management to reinvent the wheel once again after so many years of teaching.
  The effort you have to put in to really provide a user (student) friendly environment- especially where a lot of explanation is required.
  There is no way you can just 'dumb' an old lecture on BB and expect students to do all the work.
  Student interaction without faces- it is difficult to engage without student faces- although I think the students loved the collaborate sessions- They stayed on line for up to 3 h without leaving the sessions (except for the coffee break).
  Data- using my own data is challenging and the struggle with a good internet connection in my office
31. To adapt existing learning materials to the online environment—especially how time consuming it is.
  To try to maintain test security
32. To apply the more technical aspects of teaching
33. **To gauge where the students were academically and emotionally.**
  **To maintain the teacher-student bond.**
34. uncertainty

### What were your Most Positive Experiences of the Transition to Online Teaching? (N = 32 + 4 Missing)

1. It is not the same as face to face sessions
2. Actual whereabouts not important- can access from anywhere
3. **Being creative and innovative**
  Showing how resilient one can be
  Team work and support
4. Better time management working from home.
  Mastering online skills and feeling of uncertainty
5. Convenience You can give lectures from anywhere as long as you have internet
6. Convenience of working remotely
7. Developing new skills and learning methods
8. Development of new or revised teaching materials.
  Ability to engage the students in the periphery and ensure that they have been receiving similar teaching materials
9. Ease of doing presentations.
  Understanding of the student group on the importance of shifting to on-line training
10. Effect of assessments om QM and Blackboard after setting up the tests
11. I am grateful for learning the skills to use technology- it is so intimidating once you do it.
12. **I could teach from the comfort of my home. I could be contacted online anywhere at any time**
13. I prefer interactive face-to-face practical sessions (with the necessary Covid measures in place) to online learning.
  **Online learning benefits the students who are very self-motivated** to learn and **is an obstacle for those students who benefit from the sense of community that face to face learning offers**
14. If lectures are pre-recorded it frees up time especially if busy with unexpected clinical responsibilities
15. **Learning new online teaching skills- and feeling more confident and empowered in that regard**
16. **New experiences and broadening of my horizon in online teaching.**
  Assistance I receive from Kgosimang Mokhitli who responds so quickly to every query. Support from colleagues and CTL—constantly being aware that we are in this together
17. Only the students that wanted to be on live BB collab sessions were present in real time which makes the interaction better. You dont have to deal with students who are just there for the 80% attendance.
  I had the opportunity to use blackboard more extensively because it was forced by lockdown as the way to teach students
  Students managed to pass without the forced 80% attendance
18. Response of students to the discussions
19. Review of all material
  Innovative methods can be implemented
20. Saves time travelling to and from the University.
  All sessions easily recordable
21. **Students tend to receive more individual attention when using the online teaching platform** and this type of interaction is therefore mutually beneficial
22. The effort of the students- especially the first year students.
  **The 'million' emails and questions. I think it is much easier for students to ask questions behind an 'unknown identity'.**
  The extremely positive feedback via emails from the students. The outcome of the Semester I and II performance (marks
23. **The immense support offered by CTL to assist staff in creating the online platform**
24. The number of students that attended the sessions were always very high
25. The positive feedback from students and the fact that all my lectures are now done with powerpoint audio insertions which make it easy to update
26. The show could go on- was possible to cover the components of curriculum
27. To gain more flexibility
28. **Unrestricted access to the learning material**
29. Very good participation and attendance
30. **You only have to give a lecture once**
31. something new
32. amount of training made available to make this transition easier

### What are your Suggested Training Needs? (N = 28 + 8 Missing)

1. Blackboard training
2. Blackboard How to design a module
3. Clinical material- such as videos demonstrating examination techniques and case discussions that are relevant to our context are hard to come by- so these would be helpful for student training.
  As for staff training- CTL has truly done an excellent job—there is nothing I require personally
4. Complete BB refresher
5. **Continuous training in Blackboard features—I am only using a very small part of what it actually provides. I am probably not even aware of all the possibilities**
6. Developing groups and have group assignments and interactions
7. Formal training in ZOOM- Blackboard etc.
8. **I am yet to be convinced that practical clinical skills can be better taught online- rather than in- person.**
   I would appreciate training in how to manage increasing class size with the same number of staff without sacrificing on the quality of learning
9. I need further assistance in conforming my lectures to be more interactive
10. I think all the available training opportunities are sufficient to successfully- and confidently navigate the online platform. However- there is something very fulfilling about face-to-face contact sessions with students that cannot be replicated/mimicked online. I personally thrive seeing student faces light up when discussing an exciting fact related to the module content in class- seeing them interested- excited- and engaged is unfortunately an experience which one misses online. That being said- these are extraordinary times- and we can only do our best to facilitate the student learning process
11. I think we may benefit from collaborate webinars with lecturers of other universities on their online experiences in similar modules.
  Teaching and learning really went the extra mile during the transition to online teaching
12. I would like more personal interaction with blackboard personal in the way to set up online tests and assessments- the broad outlines were not helpful for me in the way that I need to evaluate the students.
  My subject does not have alot of scope for true/false or MCQ type questions and the essay type answers seems very tedious and difficult to mark
13. I would prefer more training on how to use blogs and discussion boards
14. It's not about training- it's about time
15. Methods for auditing / appraising the online tools
16. More training
17. **My modules are better learned physically than online.** I wish for the online portal to be a lesser option preferred by the university than physical
18. Need better video editing and presentations tools in order to produce more professional end more student engaging presentations contact
19. Nil
20. None
21. Presenting practical sessions and tutorials online
22. Repeated continuous training
23. Student engagement in online learning
  Creative ideas in online learning
24. To swap back to face to face for clinical teaching asap
  The feedback from students is poor of electronic platform. **Very impersonal and not conducive to training**
25. Ways to engage with students on the online platform
26. We need to get back to the classrooms
27. None
28. None thus far.

## Footnotes

1 BBC: Blackboard Collaborate.

2 MCQs: Multiple Choice Questions.

3 NSFAS: National Student Financial Aid Scheme (https://www.nsfas.org.za/content/index.html).
